# Concordant Open Neural Tube Defects in a Monochorionic Twin Pair of a Dichorionic-Triamniotic Triplet Gestation

**DOI:** 10.1055/a-2848-3193

**Published:** 2026-04-22

**Authors:** Olivia Van Benschoten, Lauryn Gabby, Shreya Visvanathan, Ashlyn Hodges, Lori Moore, Martin Olsen, Aleksandr Fuks

**Affiliations:** 1 4154 Department of Obstetrics and Gynecology, East Tennessee State University, Johnson City, United States

**Keywords:** multifetal gestation, open neural tube defects, in-utero repair, genetics

## Abstract

**Introduction** Myelomeningocele (MMC) is a type of open neural defect that involves exposure of the meninges and neural elements. There are fewer than 30 cases of twins with concordant MMCs reported in the literature. We discuss a case of dichorionic-triamniotic triplets in which the monochorionic-diamniotic pair was diagnosed antenatally with MMC.

**Case Summary** The mother presented for routine prenatal care at 11 weeks of gestation as a 31-year-old G5P4003. A limited ultrasound at 18 weeks of gestation confirmed her estimated due date, and three viable fetuses were observed. She was found to have dichorionic-triamniotic triplets with Triplet A and Triplet B sharing a placenta. A detailed anatomic survey demonstrated MMC of Triplet B at L4-S2 and a suspected open neural tube defect in Triplet A. Triplet C had no apparent defects on ultrasound. MMC of Triplets A and B was confirmed postnatally. Chromosome microarray analyses for the triplets were normal.

**Discussion** Most cases of MMC in monozygotic twins are nonconcordant, and the role of genetics remains ambiguous. Our case suggests a possible shared genetic etiology. Future studies may investigate the potential for a single-gene mutation driving the development of MMC.

## Introduction

Myelomeningocele (MMC) impacts 3 in every 10,000 live births in the United States. 1 Concordance of MMCs in the setting of twin and triplet pregnancies is an incredibly rare phenomenon, with less than 30 cases of twins with concordant MMCs having been reported in the literature. 2 To date, there are no clearly documented inherited forms of meningomyelocele, and the exact etiology of MMC remains poorly understood. The occurrence of MMCs within multifetal gestations and among families with intergenerational MMCs suggests that there could be genetic factors contributing to these conditions. 3 Twin studies reveal intriguing patterns: while MMCs in monozygotic twins would support the hypothesis that there is a genetic predisposition to this malformation, dizygotic twins demonstrate a greater number of dual MMCs, which could instead support the role of environmental exposures in the development of neural tube defects. 2 Only 5% of cases of MMC have a positive family history. Currently, genetic factors, dietary elements, and environmental exposures are the risk factors most strongly associated with this condition. 3 4 5 In this case report, we discuss a rare case of dichorionic-triamniotic triplets in which the monochorionic-diamniotic pair was both diagnosed antenatally with MMC.

## Case Summary

**Fig. 1:**
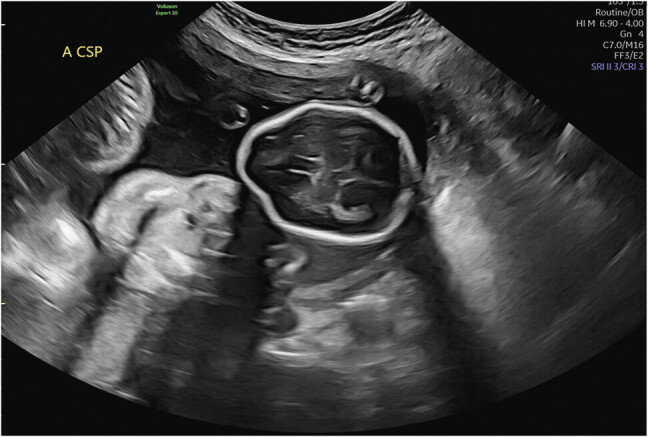
Demonstration of the lemon sign of triplet A at 22 ^5/7^ weeks of ultrasound.

**Fig. 2:**
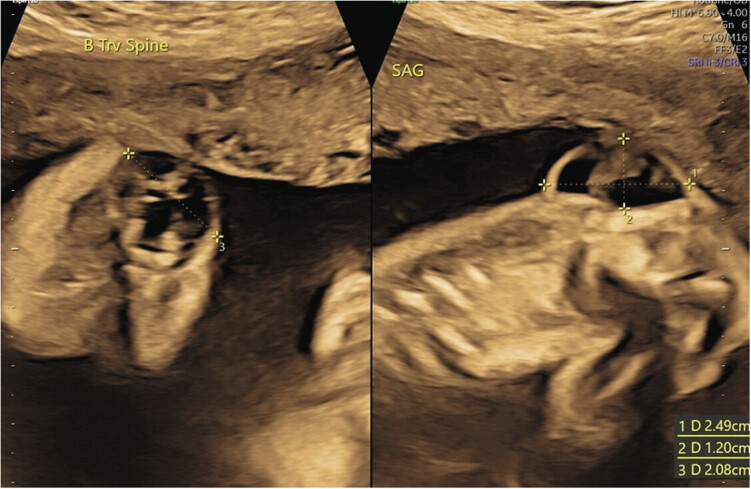
Demonstration of MMC of triplet B at 22 ^5/7^ weeks of ultrasound.

A 31-year-old G5P4003 patient presented for routine prenatal care at 11 weeks of gestation. Medical history was remarkable for ongoing tobacco use and a prior history of neonatal demise secondary to a congenital heart defect. Prepregnancy BMI was 23. She denied any history of folate deficiency, environmental exposures, teratogens, or antiepileptic medication use. She was counseled on the importance of taking an adequate folic acid supplement in early pregnancy. She took a multivitamin with 0.24 mcg folic acid daily. She did not take a prenatal-specific vitamin or folic acid supplement. A limited ultrasound at 18 ^6/7^ weeks of gestation confirmed dichorionic-triamniotic triplets with Triplet A and Triplet B sharing a placenta, while Triplet C had a separate placenta. Detailed anatomic surveys were undertaken at 22 ^5/7^ weeks of gestation, which demonstrated MMC of Triplet B at L4-S2 and a suspected open neural tube defect in Triplet A based upon scalloped frontal bones, bowing of the cerebellum, and posterior fossa collapse as seen in Figs. 1 and 2 . 6 7 Triplets A and B were also found to have mild bilateral cerebral ventriculomegaly. Triplet B was diagnosed with fetal growth restriction at that time, as well as an estimated fetal weight at the 9th percentile. Triplet C was appropriately grown and had no apparent abnormalities on ultrasound.

The patient was offered both genetic screening with noninvasive perinatal screening as well as amniocentesis at the time of these findings but declined. Initial laboratory evaluation revealed iron-deficiency anemia. Otherwise, she had normal thyroid function studies, triglyceride studies, and a normal folate level.

Further ultrasound evaluation demonstrated Chiari II malformations of both Triplet A and Triplet B, as well as bilateral talipes equinovarus of Triplet B. On an antenatal echocardiogram, both Triplets A and B had evidence of small muscular ventricular septal defects.

The patient was referred for genetic and pediatric neurosurgery consultations at a tertiary maternal–fetal care center, as well as delivery planning. Following discussion with the tertiary care center, she underwent cell-free DNA screening. The cell-free DNA screening returned low risk for the common aneuploidies. Ultrasound at 28 ^5/7^ weeks redemonstrated the lumbar MMC of Triplet A and demonstrated a sacral MMC of Triplet B. The patient was ultimately admitted to the referral center at 31 ^4/7^ weeks of gestation for fetal monitoring and antenatal corticosteroids following a new diagnosis of absent end-diastolic flow on umbilical artery Doppler studies of Triplet B. She was subsequently delivered via uncomplicated classical Cesarean section at 31 ^5/7^ weeks of gestation. Triplet A had Apgars of 5 and 8, Triplet B had Apgars of 8 and 9, and Triplet C had Apgars of 5 and 5 at 1 and 5 minutes of life, respectively.

The neonates were admitted to the neonatal intensive care unit. Triplet A was confirmed to have a lumbosacral MMC with Chiari II malformation and ventriculomegaly. She underwent MMC closure and repair on the third day of life. Serial head imaging ultimately revealed progressive macrocephaly and hydrocephalus. She was discharged home with outpatient neurosurgery follow-up and underwent endoscopic third ventriculostomy and ventriculoperitoneal shunt placement between 4 and 5 months of life. Following this surgery, she experienced seizures requiring an additional 7-day hospitalization. To date, Triplet A requires anti-epileptic drugs and follows with neurology. She was also found to have a neurogenic bladder, but does not presently require intermittent catheterization. A small to moderate-sized apical ventricular septal defect was also confirmed after delivery and has been monitored expectantly in the outpatient setting.

MMC and Chiari II malformation were also confirmed in Triplet B after delivery. She underwent repair of MMC on day of life 3. Serial head ultrasounds and MRIs showed stable mild to moderate ventriculomegaly. Triplet B also had bilateral calcaneovalgus foot deformities and two small apical ventricular septal defects with a moderate VSD shunt. Her cardiac concerns are being expectantly managed in the outpatient setting.

Triplet C was found to have a trivial anterior muscular ventricular septal defect, right ear hemangioma, and later developed intraventricular hemorrhage and laryngomalacia attributed to prematurity.

Chromosomal microarrays performed on Triplets A and B after delivery were negative for any variants. The triplets were all discharged from the NICU at 62 days of life.

## Discussion

This is the only reported case of triplets in which the genetically identical pair was similarly impacted by MMC. Ultimately, while there is insufficient understanding of its etiology, MMC appears to result from a complex multifactorial interplay of genetic and environmental factors. 3 8 9 10 A genetic predisposition is plausible, given that the recurrence risk of ONTD in siblings of an affected child is approximately 4%. That risk increases to 10% if there are two previously affected siblings. 11 12 Because most of the cases of MMC in monozygotic twins are nonconcordant, the role of genetics remains ambiguous. 13 Our case differs from this trend in that the monochorionic-diamniotic pair was both impacted, suggesting a shared single cause. Though one instance of monozygotic twins (in this set of triplets) does not prove that a single mutation can cause MMC, it raises the compelling possibility of a single gene or locus responsible for this case.

Genetic susceptibility in nonsyndromic ONTD cases could arise from several pathways. In addition to known maternal and sex-influenced effects in neural tube defects, factors such as folate metabolism, genetic variations in folate transporters, and assisted reproductive technologies like in vitro fertilization have also been hypothesized to potentially play significant roles. 14 15 Successful neurulation in early embryogenesis involves planar cell polarity signaling, crucial for initiation of closure of the neural tube, and the sonic hedgehog signaling pathway, which is critical for neural plate folding. VANGL1, VANGL2, PRICKLE1, SCRIB, DACT1, and CELSR1 are signaling genes currently under investigation. Folic acid and folate metabolism are also known to be significant modifiers of ONTD risk. Polymorphisms affecting methylene-tetrahydrofolate reductase, folate receptors (FOLR1, FOLR2), and methionine synthase are under investigation and may provide further insight into ONTD risk. 16 17 Despite extensive research into these various factors, the precise etiology of MMC remains elusive, with less than 20% of studied candidate genes demonstrating even a minor risk association, highlighting the need for more comprehensive genetic investigations as well as further evaluation of potential environmental risk factors. 8 While establishing definitive genetic causality is uncertain, employing whole exome sequencing on monozygotic twin subjects with concurrent MMC could potentially unveil underlying genetic mechanisms contributing to their condition.

Since a landmark randomized clinical trial of prenatal versus postnatal repair of MMC in 2010, in-utero repair of MMC has become an increasingly accepted practice and may confer significant developmental advantages to the impacted infant. 13 Multifetal gestations have not been included in the MMC repair trials due to potential harm to the unaffected fetus or fetuses. 18 However, our case raises the interesting question of in-utero repair for pregnancies in which both or all fetuses are impacted by MMC. Though potential harm (i.e., increased risk of preterm delivery) existed for the unaffected triplet, should in-utero repair have been attempted in this case, there may be future multifetal gestations in which all fetuses are impacted by MMC. Future studies may investigate the feasibility of in-utero repair of MMC for multifetal gestations as well as the potential for single-gene mutations driving the development of MMC.

## Data Availability

Data supporting the aforementioned findings of this case report are available from the corresponding author upon request.
